# Risks and Prognoses of Alzheimer's Disease and Vascular Dementia in Patients With Insomnia: A Nationwide Population-Based Study

**DOI:** 10.3389/fneur.2021.611446

**Published:** 2021-05-07

**Authors:** Min Seok Baek, Kyungdo Han, Hyuk-Sung Kwon, Yong-ho Lee, Hanna Cho, Chul Hyoung Lyoo

**Affiliations:** ^1^Department of Neurology, Gangnam Severance Hospital, Yonsei University College of Medicine, Seoul, South Korea; ^2^Department of Neurology, Wonju Severance Christian Hospital, Yonsei University Wonju College of Medicine, Seoul, South Korea; ^3^Department of Statistics and Actuarial Science, Soongsil University, Seoul, South Korea; ^4^Department of Internal Medicine, Severance Hospital, Yonsei University College of Medicine, Seoul, South Korea

**Keywords:** Alzheimer's disease, vascular dementia, insomnia, prevalence, mortality, prognosis

## Abstract

This study aimed to investigate the risk and prognosis of Alzheimer's disease (AD) and vascular dementia (VaD) in patients with insomnia using the National Health Insurance Service database covering the entire population of the Republic of Korea from 2007 to 2014. In total, 2,796,871 patients aged 40 years or older with insomnia were enrolled, and 5,593,742 controls were matched using a Greedy digit match algorithm. Mortality and the rate of admission to a long-term care facility were estimated using multivariable Cox analysis. Of all patients with insomnia, 138,270 (4.94%) and 26,706 (0.96%) were newly diagnosed with AD and VaD, respectively. The incidence rate ratios for AD and VaD were 1.73 and 2.10, respectively, in patients with insomnia compared with those without. Higher mortality rates and long-term care facility admission rates were also observed in patients with dementia in the insomnia group. Known cardiovascular risk factors showed interactions with the effects of insomnia on the risk of AD and VaD. However, the effects of insomnia on the incidence of AD and VaD were consistent between the groups with and without cardiovascular risk factors. Insomnia is a medically modifiable and policy-accessible risk factor and prognostic marker of AD and VaD.

## Introduction

Dementia is a progressive neurodegenerative disorder characterized by impairment in cognition that interferes with daily life activities (1). Alzheimer's disease (AD) and vascular dementia (VaD) are the most prevalent causes of dementia (2). Dementia is a burden on both patients and caregivers, and also increases the global socioeconomic burden (3). To effectively counter these burdens, a number of national policies have focused on the management and prevention of the risk factors associated with dementia.

Insomnia is characterized by difficulty in sleep initiation or maintenance with compromised daytime function (4). Insomnia has been reported to be related to the pathologic hallmark proteins of AD, amyloid-β (Aβ). Deposition of Aβ was caused by inhibition of clearance pathway, and increased synaptic activity that facilitate the accumulation of Aβ (5, 6). In positron-emission tomography (PET) imaging study, poor quality of sleep was associated with a greater cortical Aβ burden in cognitively normal elderly (7, 8). Furthermore, insomnia was associated with the cerebrovascular disease that increase the risk of VaD through disrupting subcortical circuits, which are related to the sleep cycle (9–11).

Recently, two studies conducted in Taiwan investigated the relationship between the incidence of insomnia and dementia using data sampled from national medical records and found a higher risk of dementia in patients with insomnia (12, 13). However, despite the large cohort sizes of over 50,000 patients with insomnia in these studies, analyses of population data samples may be limited by sampling bias or systematic errors (14). In addition, while several studies have investigated the risk of dementia in patients with insomnia, to the best of our knowledge, only a limited number of studies have investigated the effect of insomnia on the prognosis of dementia.

In this study, the effects of insomnia on incident AD and VaD were investigated using a large dataset encompassing the medical records of the entire national population of the Republic of Korea. The prognosis of AD and VaD were ascertained by comparing mortality records and long-term care facility admission records between patients with insomnia and those without insomnia. Finally, the association between insomnia and known cardiovascular risks was analyzed to establish the direct effects of insomnia on AD and VaD incidence.

## Materials and Methods

### Participants

This study was a retrospective nationwide population-based cohort study, using data from a national health insurance claims database established by the National Health Insurance Service (NHIS) of Korea. The NHIS is a mandatory health insurance system in the Republic of Korea, which encompassed 97.3% (52 million) of the Korean population, as of 2015. Since the adoption of the fee-for-service model by the NHIS to pay healthcare providers, additional information such as the history of diagnosis codes, details of medical bills, drug prescription and procedures, and demographic records have been compiled. Health Insurance Review Agency (HIRA) provides a quality control role and evaluates healthcare performance and reviews medical data (15). This database is provided by the “Big Data Steering Department” of the NHIS for research purposes and encompasses information on the entire Korean population. Further details regarding this database have been provided previously (16).

We defined the diagnoses of insomnia, AD, and VaD using the tenth revision of the International Statistical Classification of Diseases (ICD-10) codes recorded in the NHIS database. Individuals were defined as having insomnia if the code G47.0 (disorders of initiating and maintaining sleep) was recorded in the NHIS database. The diagnosis of AD was defined by having the code F00 (Dementia in Alzheimer disease) or G30 (Alzheimer disease) in the NHIS database. The diagnosis of VaD was defined by having the code F01 (Vascular dementia). The date of dementia diagnosis was defined as the date when the prescription of an anti-dementia medication and registration of a dementia code coincided. The diagnostic criteria for dementia based on ICD-10 codes have been reported in previous studies (17, 18).

**Figure 1:**
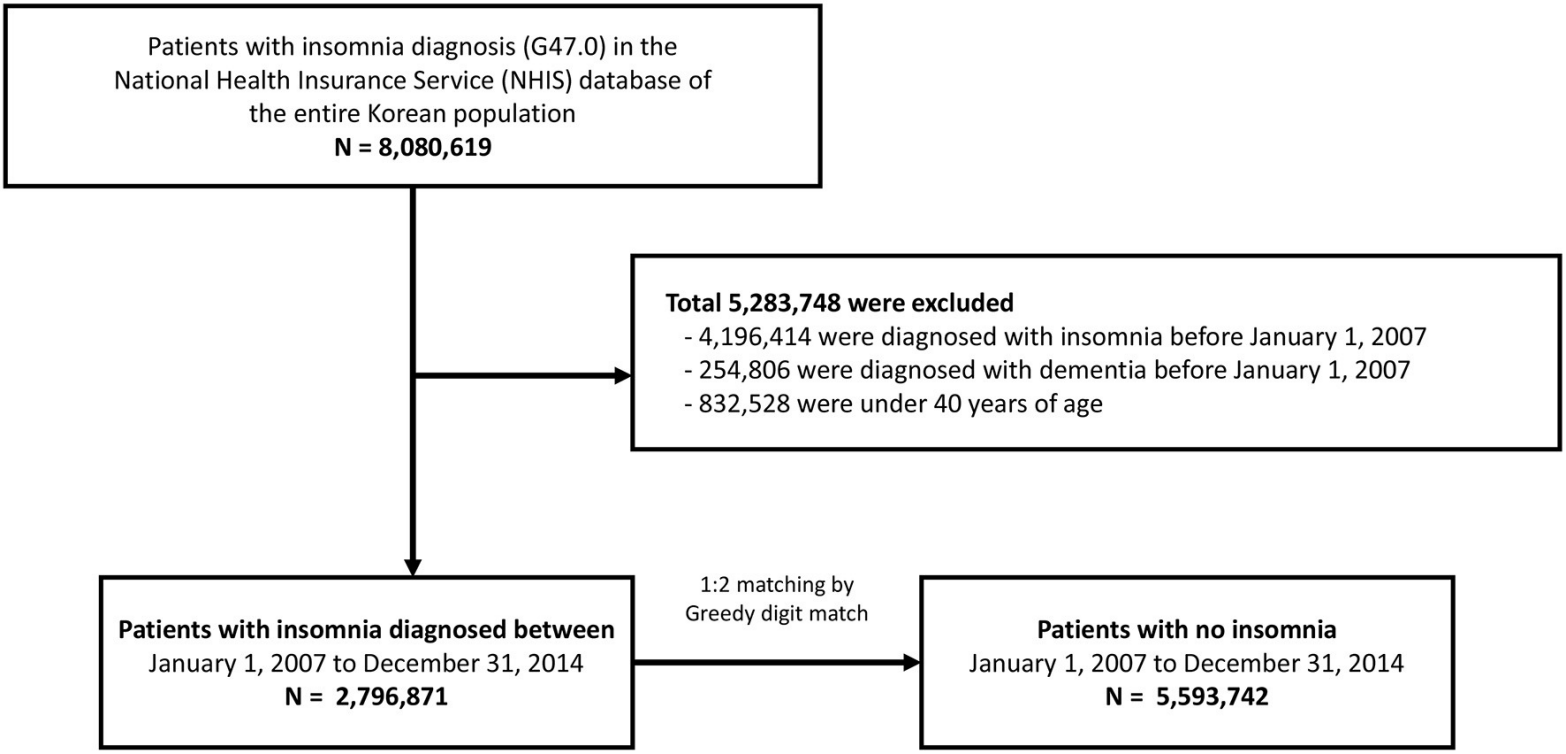
Flowchart of the study design.

Among the entire national population, 8,080,619 patients diagnosed with insomnia between 2007 and 2014 were included in this study. A total of 4,196,414 patients and 254,806 patients who were initially diagnosed with insomnia and dementia, respectively, before 2007 were excluded, and 832,528 patients aged <40 years were excluded. Following this, the insomnia group included 2,796,871 patients (Figure 1). We used the propensity score analysis to generate the age- and sex- matching control group. The demographic confounders (low income, and place of residence), and medical histories (hypertension, diabetes mellitus, dyslipidemia, congestive heart failure, ischemic heart disease, atrial fibrillation, chronic obstructive pulmonary disease, and ischemic stroke) were adjusted in the analysis. Finally, 1:2 matching without replacements was conducted using a Greedy digit match algorithm, yielding 5,593,742 individuals in the non-insomnia group. For all participants, the number of AD and VaD diagnoses as well as information on mortality and long-term care facility admission were analyzed.

This study was approved by the NHIS inquiry commission and the institutional review board of Gangnam Severance Hospital (IRB-No 3-2016-0307). The requirement for informed consent was waived by institutional review board which approved the study. The privacy of each participant was protected by de-identification of the national insurance claims data for analysis. All research was performed in accordance with the relevant guidelines and regulations.

### Definition

Patients with hypertension were indicated when anti-hypertensive medications were prescribed with ICD-10 codes of I10 (essential hypertension), I13 (hypertensive heart and renal disease), or I15 (secondary hypertension). Patients were defined as having type 2 diabetes if anti-diabetic drugs (insulins, sulfonylureas, metformin, meglitinides, thiazolidinediones, dipeptidyl peptidase-4 inhibitors, and α-glucosidase inhibitors) were prescribed with ICD-10 codes of E11 (non-insulin-dependent diabetes mellitus), E12 (malnutrition-related diabetes mellitus), E13 (other specified diabetes mellitus), or E14 (unspecified diabetes mellitus). Patients were defined as having dyslipidemia by the presence of the ICD-10 code E78 (disorders of lipoprotein metabolism and other lipidemias).

The ICD-10 codes used for other cardiovascular risks were as follows. The ICD-10 codes for ischemic heart disease (IHD) are I20 (angina pectoris), I21 (ST elevation and non-ST elevation myocardial infarction), I22 (subsequent ST elevation and non-ST elevation myocardial infarction), I23 (current complications following ST elevation and non-ST elevation myocardial infarction), I24 (other acute ischemic heart diseases), and I25 (chronic ischemic heart disease); for stroke, the ICD-10 codes are I63 (cerebral infarction) and I64 (stroke, not specified as hemorrhage or infarction); for atrial fibrillation (AF), the ICD-10 code is I48.0 (atrial fibrillation and flutter); for congestive heart failure (CHF), the ICD-10 code is I50.0 (congestive heart failure); and for chronic obstructive pulmonary disease, (COPD) the ICD-10 codes are J41 (simple and mucopurulent chronic bronchitis), J42 (unspecified chronic bronchitis), J43 (emphysema), and J44 (other chronic obstructive pulmonary disease). The detailed list of ICD-10 codes for diagnoses is presented in Supplementary Table 1.

### Statistical Analysis

For acquiring the propensity score-matched cohort, categorical and continuous variables were compared using the McNemar's test and paired *t*-test, respectively. The cumulative event rates were compared between the insomnia and non-insomnia groups based on Kaplan-Meier censoring estimates, using the log-rank test. Crude risks were analyzed in the overall cohort, and the adjusted incidence rate ratio (IRR) was subsequently estimated based on the results of multivariable Cox regression analyses after adjustment for age and sex. Statistical analyses were performed using SAS version 9.3 (SAS Institute, Cary, North Carolina, USA), Stata statistical software, release 12 (StataCorp, College Station, Texas, USA), and SPSS statistical package, version 19.0 (SPSS Inc., Chicago, IL, USA).

## Results

### Population Characteristics

The baseline characteristics of participants are presented in Table 1. The numbers of individuals in the insomnia and non-insomnia groups were 2,796,871 and 5,593,742, respectively. The mean age was 59.48 years, and 37.69% were male in both groups. The proportions of cardiovascular risks factors, such as diabetes mellitus, hypertension, dyslipidemia, stroke, IHD, CHF, AF, and COPD were higher in the insomnia group (Table 1).

**Table 1:** Baseline characteristics of patients.

	**Insomnia**	
	**No**	**Yes**
*N*	5,593,742	2,796,871
Number of patient-years	26,305,394	12,319,096
Age (years)	59.48 ± 11.82	59.48 ± 11.82
Male (%)	2,108,552 (37.7)	1,054,276 (37.7)
Diabetes mellitus (%)	596,889 (10.7)	421,342 (15.1)
Hypertension (%)	1,664,606 (29.8)	1,106,869 (39.6)
Dyslipidemia (%)	870,731 (15.6)	643,958 (23.0)
Stroke (%)	21,738 (0.4)	50,348 (1.8)
Ischemic heart disease (%)	426,884 (7.6)	415,221 (14.9)
Congestive heart failure (%)	20,226 (0.4)	44,209 (1.6)
Atrial fibrillation (%)	51,944 (0.9)	54,172 (1.9)
Chronic obstructive pulmonary disease (%)	439,469 (7.9)	434,583 (15.5)

*Data are presented as mean ± SD or number (percentage)*.

### Incidence Rates of Alzheimer's Disease and Vascular Dementia in Patients With Insomnia

Of the 5,593,742 individuals in the non-insomnia group, 160,660 (2.87%) and 25,058 (0.45%) individuals were newly diagnosed with AD and VaD during 26,305,394 person-years, respectively. The crude incidence rate was 6.11 per 1,000 person-years for AD and 0.95 per 1,000 person-years for VaD. In the insomnia group, 138,270 (4.94%) and 26,706 (0.96%) individuals were correspondingly diagnosed with AD and VaD during 12,319,096 person-years, respectively. The crude incidence rates of AD and VaD were 11.22 per 1,000 person-years and 2.17 per 1,000 person-years, respectively (Table 2).

**Table 2:** Incidence rate of dementia in the insomnia and non-insomnia groups.

			**Alzheimer's disease**				**Vascular dementia**			
	**Insomnia**	***N***	**Events**	**Person-Years**	**Crude IR^†^**	**IRR (95% CI)^‡^**	**Events**	**Person-Years**	**Crude IR^†^**	**IRR (95% CI)^‡^**
Total	No	5,593,742	160,660	26,305,394	6.11	1 (ref.)	25,058	26,305,394	0.95	1 (ref.)
	Yes	2,796,871	138,270	12,319,096	11.22	1.73 (1.72, 1.75)	26,706	12,319,096	2.17	2.10 (2.06, 2.14)
**Sex**										
Male	No	2,108,552	45,049	9,577,665	4.70	1 (ref.)	8,575	9,577,665	0.90	1 (ref.)
	Yes	1,054,276	43,302	4,324,631	10.01	1.99 (1.96, 2.01)	10,779	4,324,631	2.49	2.53 (2.46, 2.61)
Female	No	3,485,190	115,611	16,727,729	6.91	1 (ref.)	16,483	16,727,729	0.99	1 (ref.)
	Yes	1,742,595	94,968	7,994,465	11.88	1.63 (1.61, 1.64)	15,927	7,994,465	1.99	1.88 (1.84, 1.93)
**Age**										
40–49	No	1,342,522	670	6,733,805	0.10	1 (ref.)	365	6,733,805	0.05	1 (ref.)
	Yes	671,261	1,585	3,282,546	0.48	4.64 (4.24, 5.08)	987	3,282,546	0.30	5.03 (4.45, 5.68)
50–59	No	1,616,552	4,458	7,655,274	0.58	1 (ref.)	1,415	7,655,274	0.19	1 (ref.)
	Yes	808,276	7,017	3,688,638	1.90	3.12 (3.00, 3.24)	2,843	3,688,638	0.77	3.84 (3.60, 4.10)
60–69	No	1,354,602	30,120	6,545,914	4.60	1 (ref.)	5,941	6,545,914	0.91	1 (ref.)
	Yes	677,301	31,835	3,049,836	10.44	2.19 (2.16, 2.23)	7,130	3,049,836	2.34	2.43 (2.34, 2.51)
70–79	No	994,826	81,522	4,363,196	18.68	1 (ref.)	12,352	4,363,196	2.83	1 (ref.)
	Yes	497,413	65,928	1,905,019	34.61	1.77 (1.75, 1.79)	11,438	1,905,019	6.00	1.97 (1.92, 2.02)
80–89	No	262,792	40,836	947,601	43.10	1 (ref.)	4,735	947,601	5.00	1 (ref.)
	Yes	131,396	29,458	372,652	79.05	1.65 (1.63, 1.68)	4,027	372,652	10.81	1.92 (1.84, 2.01)
>90	No	22,448	3,054	59,605	51.24	1 (ref.)	250	59,605	4.20	1 (ref.)
	Yes	11,224	2,447	20,405	119.92	1.86 (1.75, 1.97)	281	20,405	13.77	2.56 (2.13, 3.07)

† *Crude incidence rate of dementia per 1,000 person-years*.

‡ *Incidence rate ratio adjusted for age and sex*.

*IR, incidence rate; IRR, incidence rate ratio; CI, confidence interval; ref, reference*.

**Figure 2:**
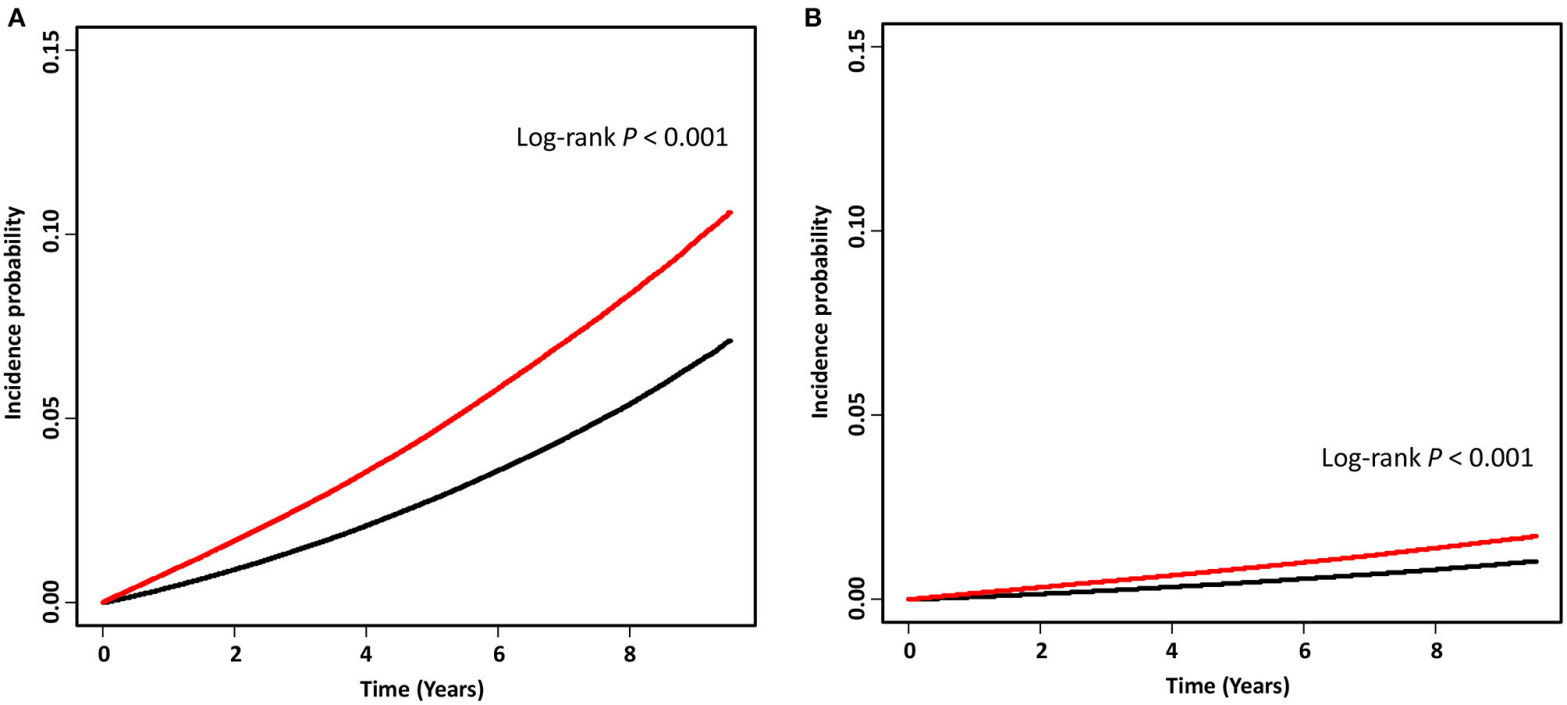
Kaplan-Meier curves of the incidence probability of Alzheimer's disease **(A)** and vascular dementia **(B)** in the insomnia group (red) and non-insomnia group (black). A wash-out period of 6 months was used to minimize the possibility of reverse-causality. Therefore, time “0” was set as the time point 6 months after insomnia diagnosis.

The IRRs of AD and VaD in the insomnia group were 1.73 (1.72–1.75, 95% CI) and 2.10 (2.06–2.14, 95% CI), respectively (Table 2). Higher IRR values for AD and VaD were observed in men than in women, and the younger age group tended to show higher IRRs for AD and VaD. However, the IRR in those aged ≥90 years was higher than that in those in their 80s. The cumulative incidence changes also showed the higher incidence of AD and VaD in the insomnia group (Figure 2).

### Long-Term Care Facility Admission and Mortality in the Insomnia and Non-insomnia Groups

Among patients with AD in the insomnia group, the rate of admission to long-term care facilities was 26.6 per 1,000 person-years, a value 1.2-fold higher than that in patients with AD in the non-insomnia group. Among patients with VaD in the insomnia group, 26.3 patients per 1,000 person-years were admitted to long-term care facilities, and the age- and sex-adjusted hazard ratio was 1.21 compared to the patients with VaD in the non-insomnia group (Table 3). In addition, patients with AD and VaD in the insomnia group had higher mortality rates than those in the non-insomnia group (Table 3).

**Table 3:** Rates of long-term care facility admission and mortality from dementia in the insomnia and non-insomnia groups.

	**Insomnia**	**Admission rate (/1,000 person-years)**	**Adjusted HR (95% CI)**	**Mortality rate (/1,000 person-years)**	**Adjusted HR (95% CI)**
Alzheimer's disease	No	21.0	1 (ref.)	129.3	1 (ref.)
	Yes	26.6	1.24 (1.21, 1.28)	133.5	1.07 (1.04, 1.10)
Vascular dementia	No	21.2	1 (ref.)	131.4	1 (ref.)
	Yes	26.3	1.21 (1.13, 1.30)	128.2	1.08 (1.01, 1.15)

*HR, Hazard ratio; CI, confidence interval; ref, reference*.

### Interaction of Insomnia With the Cardiovascular Risks

The IRR of AD in the insomnia group was lower in patients with underlying cardiovascular risks, such as diabetes mellitus, dyslipidemia, stroke, IHD, CHF, AF, and COPD than in those without. These cardiovascular risk factors showed interactions with the effects of insomnia on the risk of AD (interaction ***P*** < *0.001*, Table 4). However, the IRRs of AD in patients with or without cardiovascular risks were still higher than those in the non-insomnia groups (IRR > 1.0, Table 4). In the VaD patients with insomnia, diabetes mellitus, stroke, IHD, and CHF showed an interaction with the effects of insomnia on the risk of VaD (Table 4).

**Table 4:** Subgroup analysis for dementia risk in patients with insomnia.

	**Alzheimer's disease**				**Vascular dementia**			
	**IRR**	**Lower**	**Upper**	**Interaction *P***	**IRR**	**Lower**	**Upper**	**Interaction *P***
DM	1.59	1.56	1.61	<0.001	1.81	1.75	1.88	<0.001
Without DM	1.70	1.68	1.71		2.07	2.03	2.11	
HTN	1.59	1.57	1.60	0.371	1.90	1.85	1.94	0.057
Without HTN	1.57	1.55	1.59		1.84	1.78	1.89	
Dyslipidemia	1.61	1.59	1.64	<0.001	1.99	1.92	2.06	0.488
Without dyslipidemia	1.71	1.70	1.73		2.01	1.97	2.06	
Stroke	1.18	1.13	1.24	<0.001	1.48	1.38	1.58	<0.001
Without stroke	1.69	1.67	1.70		1.92	1.88	1.96	
IHD	1.56	1.53	1.58	<0.001	1.80	1.74	1.88	<0.001
Without IHD	1.64	1.62	1.65		1.98	1.94	2.02	
CHF	1.24	1.18	1.31	<0.001	1.53	1.36	1.73	<0.001
Without CHF	1.70	1.69	1.71		2.05	2.02	2.09	
AF	1.53	1.47	1.60	<0.001	1.96	1.80	2.13	0.734
Without AF	1.72	1.70	1.73		2.06	2.02	2.09	
COPD	1.58	1.56	1.61	<0.001	1.91	1.83	1.99	0.116
Without COPD	1.64	1.62	1.65		2.00	1.96	2.04	

*The Incidence rate ratio (IRR) of Alzheimer's disease (A) and vascular dementia (B) in subgroups with vascular risk factors are shown with 95% confidence interval. The effects of insomnia on the risk of dementia were weaker in subgroups with well-known cardiovascular risk factors than those without*.

*IRR, incidence rate ratio; DM, diabetes mellitus; HTN, hypertension, IHD, ischemic heart disease; CHF, congestive heart failure; AF, Atrial fibrillation; COPD, Chronic obstructive pulmonary disease; CI, confidence interval*.

## Discussion

In this study using medical data of the national population, both incidence rates and changes in the cumulative incidence of AD and VaD were found to be greater in individuals with insomnia than in those without. Moreover, worse prognosis was observed in patients with AD or VaD in the insomnia group with regard to higher rates of admission to long-term care facilities and higher mortality rates.

The role of sleep in the accumulation of Aβ has been identified in previous studies (19, 20). In amyloid precursor protein transgenic (Tg2576) mice, the Aβ burden in the cerebral interstitial fluid decreased during sleep; however, it increased while waking. This fluctuation in the levels of cerebrospinal fluid Aβ was also observed in humans (21). Previous PET studies showed that poorer quality of sleep was associated with a greater cortical Aβ burden in cognitively normal elderly (7, 8). Moreover, insomnia could be associated with an increase in the level of the sleep cycle-related hormone, orexin, and a systemic inflammation process through the activation of microglial cells, thereby resulting in Aβ accumulation (21, 22). Previously, a 1.2- to 2.3-fold increase in the risk of AD in patients with insomnia has been reported by both community-based and institutional cohort studies (23–25). Our findings using nationwide population data support the findings of previous studies; we reported a 1.7-fold higher IRR of AD in patients with insomnia. In addition, the cumulative incidence of AD was higher among patients with insomnia than among those without insomnia (Figure 2). In both the insomnia and non-insomnia groups, women showed a higher crude incidence rate of AD (Table 2). However, the IRRs of AD in patients with insomnia were higher in men than in women suggesting that insomnia may have a stronger effect on AD in men. Differences in sleeping patterns with respect to sex, especially an increase in the frequency of sleep apnea, may modulate the susceptibility to the effects of insomnia (26, 27). Furthermore, differences in orexin expression and sensitivity to the hormone, as suggested in animal studies, could explain the sex differences in the effect of insomnia on AD pathophysiology (28, 29).

Imaging studies have previously shown that insomnia is directly associated with the brain imaging biomarkers of VaD, such as white matter lesions and cerebral microbleeds (9–11). Changes in cerebral blood perfusion and systemic inflammation due to insomnia may promote small vessel disease and ischemic stroke, thereby increasing the risk of VaD (30–34). Although previous studies have observed trends for a higher risk of VaD in patients with insomnia, these were not statistically significant, possibly due to the small sample sizes (25, 35). However, an ~2-fold higher incidence of VaD in patients with insomnia was observed in the current study using nationwide population data. The interaction with underlying cardiovascular risks may have affected the results. Table 4 showed the interactions of the cardiovascular risks with the incidence of AD and VaD. The lower IRR in those with cardiovascular risk factors than in those without reflected the relatively smaller effect of insomnia on the incidence of dementia. However, consistently high IRR (>1.0) of the insomnia patients with the cardiovascular risks suggested that the effects of insomnia on the incidence of dementia is considerable, irrespective of the coexistence of cardiovascular risks (Table 4). Therefore, the effects of insomnia on the incident dementia in the patients with cardiovascular risks should not be overlooked in the clinical situations.

In addition to its effect on the incidence of AD and VaD, insomnia also influences the prognosis following dementia diagnosis. The major outcome events of dementia were expressed in terms of the rate of admission to a long-term care facility and mortality rate (36–38). In elderly people, the presence of insomnia increased the risk of long-term care facility admission due to higher occurrence of behavioral and psychological symptoms as well as burden on caregivers (39). Higher mortality and rates of admission to long-term care facilities suggest that worse prognosis can be expected in patients with pre-existing insomnia at the time of dementia diagnosis. Moreover, further investigations are required to confirm the possible modification of the dementia prognosis by clinical interventions for insomnia.

This study has several limitations. First, this is a retrospective analysis. Therefore, the results are particularly susceptible to the effects of reverse causality bias. To overcome this issue, we limited the participants to the newly diagnosed insomnia patients after 2007, and we collected data on dementia diagnosis at least 6 months after the diagnosis of insomnia for the cumulative incidence analysis to minimize bias (Figure 2). Second, dementia, insomnia, and cardiovascular risks were diagnosed based on diagnostic codes without the evaluation of medical history or neuropsychological tests. The patients who suffered from insomnia may not visit clinics or hospitals. There are also possible misdiagnoses of insomnia in the patients having major psychiatric diseases. Therefore, the incidence of insomnia could be underestimated or overestimated to the actual levels. Although alcohol consumption, smoking and obesity are reported as modifiable and important cardiovascular risks (40, 41), these information were limited in NHIS database. Thus, there could be possible discrepancies with regard to diagnosis, cardiovascular risks and medication between the real-world situation and the claims database. Also, diagnosis of mixed dementia (AD + VaD), which accounts for a significant portion of dementia cases showing multifactorial features, was not accessed. This is an inevitable limitation of research using big databases with diagnostic codes. Lastly, the effects of medication used by patients with insomnia was not considered in this study, although the relationship between medication use and the incident dementia is unclear. Exposure to benzodiazepines was reported to increase the risk of dementia (42, 43), while a recent study found that the results were related to reverse causality and confounders (44). One study showed no correlation between antidepressants use and the risk of dementia (45). Moreover, the other study found the effect of selective serotonin-reuptake inhibitor (SSRI) treatment on the delayed progression in AD (46). In addition, antihistamines in previous showed low association with the risk of dementia (47, 48). Therefore, further longitudinal analysis between medication user and non-user in the national population would clarify the effect of medication on the risk of dementia.

In conclusion, insomnia increases the incidence of AD and VaD, and it may have effects on the prognosis of dementia. As it is a medically modifiable and policy-accessible risk factor of dementia, effective strategies for insomnia may be a manageable target for preventing dementia.

## Data Availability Statement

The raw data supporting the conclusions of this article will be made available by the authors, without undue reservation.

## Ethics Statement

The studies involving human participants were reviewed and approved by the NHIS inquiry commission and the institutional review board of Gangnam Severance Hospital (IRB-No 3-2016-0307). Written informed consent from the participants' legal guardian/next of kin was not required to participate in this study in accordance with the national legislation and the institutional requirements.

## Author Contributions

MB contributed to the conception and design of the study, collection, assembly, analysis and interpretation of data, and manuscript writing. KH contributed to the conception and design of the study, collection and assembly of data, and data analysis and interpretation. H-SK and Y-hL contributed to the data analysis and interpretation. HC contributed to the study conception and design, administrative support, collection and assembly of data, and data analysis and interpretation. CL contributed to the conception and design of the study, administrative support, manuscript writing, and final approval of manuscript. All authors read and approved the final manuscript.

## Conflict of Interest

The authors declare that the research was conducted in the absence of any commercial or financial relationships that could be construed as a potential conflict of interest.
