# Supplementary material for: Risks and Prognoses of Alzheimer's Disease and Vascular Dementia in Patients With Insomnia: A Nationwide Population-Based Study
Source: Front Neurol. 2021 May 7;12:611446. doi: 10.3389/fneur.2021.611446 (PMC8137901; doi:10.3389/fneur.2021.611446)
Supplement: Supplementary file 1 [file Table_1.DOCX]

Supplementary Table 1. List of ICD-10 codes for diagnoses

| **Diagnosis** | **ICD-10 codes** |
| --- | --- |
| Insomnia | G47.0 (disorders of initiating and maintaining sleep) |
| Alzheimer's Disease | F00 (Dementia in Alzheimer disease) |
|  | G30 (Alzheimer disease) |
| Vascular Dementia | F01 (Vascular dementia) |
| Hypertension | I10 (essential hypertension) |
|  | I13 (hypertensive heart and renal disease) |
|  | I15 (secondary hypertension) |
| Diabetes Mellitus | E11 (non-insulin-dependent diabetes mellitus) |
|  | E12 (malnutrition-related diabetes mellitus) |
|  | E13 (other specified diabetes mellitus) |
|  | E14 (unspecified diabetes mellitus) |
| Dyslipidemia | E78 (disorders of lipoprotein metabolism and other lipidemias) |
| Ischemic heart disease | I20 (angina pectoris) |
|  | I21 (ST elevation and non-ST elevation myocardial infarction) |
|  | I22 (subsequent ST elevation and non-ST elevation myocardial infarction) |
|  | I23 (current complications following ST elevation and non-ST elevation myocardial infarction) |
|  | I24 (other acute ischemic heart diseases) |
|  | I25 (chronic ischemic heart disease) |
| Stroke | I63 (cerebral infarction) |
|  | I64 (stroke, not specified as hemorrhage or infarction) |
| Congestive heart failure | I50.0 (congestive heart failure) |
| Atrial fibrillation | I48.0 (atrial fibrillation and flutter) |
| Chronic obstructive pulmonary disease | J41 (simple and mucopurulent chronic bronchitis) |
|  | J42 (unspecified chronic bronchitis) |
|  | J43 (emphysema) |
|  | J44 (other chronic obstructive pulmonary disease) |
